# Development and implementation of the first national data quality standards for population-based birth defects surveillance programs in the United States

**DOI:** 10.1186/s12889-015-2223-2

**Published:** 2015-09-19

**Authors:** Marlene Anderka, Cara T. Mai, Paul A. Romitti, Glenn Copeland, Jennifer Isenburg, Marcia L. Feldkamp, Sergey Krikov, Russel Rickard, Richard S. Olney, Mark A. Canfield, Carol Stanton, Bridget Mosley, Russell S. Kirby

**Affiliations:** Massachusetts Department of Public Health, 250 Washington St. 5th floor, Boston, MA 02108 USA; National Center on Birth Defects and Developmental Disabilities, Centers for Disease Control and Prevention, Atlanta, GA USA; Department of Epidemiology, College of Public Health, The University of Iowa, Iowa City, IA USA; Michigan Department of Community Health, Lansing, MI USA; Carter Consulting, Atlanta, GA USA; Division of Medical Genetics, Department of Pediatrics, University of Utah School of Medicine, Salt Lake City, UT USA; National Birth Defects Prevention Network, Houston, TX USA; Texas Department of State Health Services, Birth Defects Epidemiology and Surveillance Branch, Austin, TX USA; Colorado Department of Public Health and Environment, Denver, CO USA; College of Medicine, University of Arkansas for Medical Sciences, Arkansas Children’s Hospital Research Institute, Little Rock, AR USA; Department of Community and Family Health, College of Public Health, University of South Florida, Tampa, FL USA

## Abstract

**Background:**

Population-based birth defects surveillance is a core public health activity in the United States (U.S.); however, the lack of national data quality standards has limited the use of birth defects surveillance data across state programs. Development of national standards will facilitate data aggregation and utilization across birth defects surveillance programs in the U.S.

**Methods:**

Based on national standards for other U.S. public health surveillance programs, existing National Birth Defects Prevention Network (NBDPN) guidelines for conducting birth defects surveillance, and information from birth defects surveillance programs regarding their current data quality practices, we developed 11 data quality measures that focused on data completeness (*n* = 5 measures), timeliness (*n* = 2), and accuracy (*n* = 4). For each measure, we established tri-level performance criteria (1 = rudimentary, 2 = essential, 3 = optimal). In January 2014, we sent birth defects surveillance programs in each state, District of Columbia, Puerto Rico, Centers for Disease Control and Prevention (CDC), and the U.S. Department of Defense Birth and Infant Health Registry an invitation to complete a self-administered NBDPN Standards Data Quality Assessment Tool. The completed forms were electronically submitted to the CDC for analyses.

**Results:**

Of 47 eligible population-based surveillance programs, 45 submitted a completed assessment tool. Two of the 45 programs did not meet minimum inclusion criteria and were excluded; thus, the final analysis included information from 43 programs. Average scores for four of the five completeness performance measures were above level 2. Conversely, the average scores for both timeliness measures and three of the four accuracy measures were below level 2. Surveillance programs using an active case-finding approach scored higher than programs using passive case-finding approaches for the completeness and accuracy measures, whereas their average scores were lower for timeliness measures.

**Conclusions:**

This initial, nation-wide assessment of data quality across U.S. population-based birth defects surveillance programs highlights areas for improvement. Using this information to identify strengths and weaknesses, the birth defects surveillance community, working through the NBDPN, can enhance and implement a consistent set of standards that can promote uniformity and enable surveillance programs to work towards improving the potential of these programs.

**Electronic supplementary material:**

The online version of this article (doi:10.1186/s12889-015-2223-2) contains supplementary material, which is available to authorized users.

## Background

Collectively, major structural birth defects are a common, costly, and critical public health challenge. In the United States (U.S.), one in every 33 babies is born with at least one of these birth defects, and one in five infants will die in their first year of life as a result of their birth defect [1, 2]. Timely and accurate population-based data on birth defects can contribute to early identification of environmental concerns, determination of etiologic agents, evaluation of prevention programs, estimation of prevalence, assessment of disparities, and timely referral to services for those with birth defects, with the hope of improving outcomes.

Although the U.S. system of national birth registration serves as an important data source for a number of health indicators, the information collected on birth defects is limited with both low sensitivity and specificity [3–5]. Instead, birth defect data in the United States are obtained from state or sub-state population-based birth defects surveillance programs [6].

A major challenge to using birth defects surveillance data effectively on a national level has been the lack of core data quality standards across birth defects surveillance programs. This lack of uniformity across programs limits the number of surveillance programs that can provide high quality data to produce national prevalence estimates for birth defects in the United States [7, 8].

Experience from other public health data collection programs in the United States, such as vital statistics or cancer, demonstrates the positive impact of establishing standards [9, 10]. Comparable data on live births, deaths, and stillbirths as well as cancer incidence, are available at the state and national level as the result of concerted collaborative efforts among all states and with strong involvement and financial support from our federal government.

As an initial step toward standards for birth defects surveillance in the United States, the National Birth Defects Prevention Network (NBDPN) published guidelines for conducting birth defects surveillance in 2004 [11]. Although these guidelines have been instrumental in assisting states to develop or enhance the operation of their birth defects surveillance programs, the absence of national standards for birth defects surveillance with established benchmarks to improve data quality and utility has limited the application of such surveillance data to improve the health of affected populations. To address this limitation, the NBDPN established a workgroup to develop national standards for quality and utility of population-based birth defects surveillance data in the United States. This paper focuses on our experience with development and implementation of these data quality standards.

## Methods

We planned a descriptive study to develop and test a data quality assessment tool for population-based birth defect surveillance programs.

### Development of the data quality assessment tool

The NBDPN Standards Workgroup queried existing birth defects surveillance programs regarding their data quality practices by contacting program managers on the NBDPN state birth defects contact list. Current standards for other public health surveillance programs (e.g., cancer registries, immunization programs, and birth defects surveillance programs in other countries) were also reviewed. The information collected, along with the existing NBDPN guidelines for conducting birth defects surveillance, provided the basis for data quality measures that focused on three attributes: completeness, timeliness, and accuracy. Completeness was defined as the extent to which all possible cases are captured and the information collected on each case is all-inclusive and comprehensive. Timeliness was defined as the extent to which case reporting or acquisition are rapid, prompt, and responsive. Lastly, accuracy was defined as the extent to which data elements are exact, correct, and valid [12].

Recognizing the variability in data collection approaches and data quality practices among U.S. population-based birth defects surveillance programs, we established tri-level performance criteria for each data quality measure. The criteria established for level 1 reflected a rudimentary level of performance by a birth defects surveillance program, those for level 2 reflected an essential level of performance, and those for level 3 represented the optimal level of performance. Our expectation was that the majority of birth defects surveillance programs in the United States should be able to achieve level 2 on all measures.

Once the criteria were established for each data quality measure, we developed a self-administered Data Quality Assessment Tool to assess a state surveillance program’s performance in data completeness, timeliness, and accuracy (Additional file 1). Early drafts of the tool were shared with selected state surveillance programs and during the 2013 NBDPN annual meeting to obtain feedback. A revised tool was piloted in June 2013 by 19 state surveillance programs who volunteered staff to complete the tool and provide comments. Additional comments were used to further refine the tool; it was finalized in January 2014 with 11 data quality measures (Table 1). Five of these measures pertained to completeness: list of birth defects monitored, pregnancy outcomes included, case identification reporting sources used, ascertainment period for case identification, and data elements collected. Two measures focused on timeliness with which case data were completed, and four measures focused on accuracy: case diagnosis verification procedures, scope of birth defects verified, level of expertise of individuals who perform case diagnosis verification, and database quality assurance process. Ad-hoc subgroups were formed to further examine the list of reportable birth defects and data elements. Finally, the self-administered tool was created as a fillable Adobe Acrobat form.

**Table 1:** National Birth Defects Prevention Network (NBDPN) levels of data quality performance measures for completeness, timeliness, and accuracy

NBDPN Data Quality (DQ) Performance Measures		Level 1	Level 2	Level 3
Completeness	DQ1.1 Types of data sources used systematically and routinely to identify potential cases at a population-based level	Each of the following sources:	The data sources in level 1 and any additional sources of natal or postnatal data	The data sources in levels 1 and 2, as well as routine reporting from any of the following data sources ^b^ for systematic specialized ascertainment of prenatally diagnosed defects.
		• Vital record data		
		• Additional source for case identification		
	DQ1.2 Birth defects included using standard NBDPN case definitions ^a^	All of the NBDPN “core” birth defects	All of the NBDPN “recommended” birth defects	Major structural malformations beyond those birth defects identified on the NBDPN list
	DQ1.3 Pregnancy outcomes included	Live births	Live births, stillbirths	Live births, stillbirths, and other pregnancy loss
	DQ1.4 Systematic and routine identification of cases (age of diagnosis) during ascertainment period	Identification of cases diagnosed through 1 month of age	Identification of cases diagnosed through 1 year of age	Identification of cases diagnosed beyond 1 year of age
	DQ1.5 Data elements collected ^a^	All “core” data elements	All “recommended” data elements	All “enhanced” data elements
Timeliness	DQ2.1 Time of case data completion for NBDPN “core” list	≥75 % of all “core” NBDPN birth defects - reported cases complete within 2 years of delivery.	≥95 % of all “core” NBDPN birth defects - reported cases complete within 2 years of delivery.	≥99 % of all “core” NBDPN birth defects - reported cases complete within 2 years of delivery.
	DQ2.2 Time of case data completion for NBDPN “recommended” list	≥75 % of all “recommended” NBDPN birth defects list-reported cases complete within 2 years of delivery.	≥95 % of all “recommended” NBDPN birth defects list-reported cases complete within 2 years of delivery.	≥99 % of a “recommended” NBDPN birth defects list-reported cases complete within 2 years of delivery.
Accuracy	DQ3.1 Data quality procedures for verification of case diagnosis	Minimal data quality procedure for case verification, majority of cases accepted as reported	Verification using “some” method, e.g., clinical case report from a specialty clinic, agreement across multiple data sources, agreement between procedure and diagnostic codes, laboratory reports	Verification using method beyond level 2, e.g., medical records
	DQ3.2 Scope of birth defects verified	Special projects, selected diagnoses, or samples only	Verification for all “core” birth defects	Verification for all “recommended” birth defects
	DQ3.3 Level of expertise for individuals who perform case diagnosis verification	Staff with no or minimal disease coding or clinical expertise perform routine case reviews	Staff with expertise in disease coding or clinical training perform routine case reviews	Clinical geneticist, dysmorphologist or other high level expert depending on defect routinely performs case reviews
	DQ3.4 Database quality assurance process	Quality checks are performed for “core” NBDPN data elements.	Quality checks are performed for “recommended” NBDPN data elements.	Quality checks are performed for “enhanced” NBDPN data elements.

^a^NBDPN list of birth defects and data elements are available at www.nbdpn.org

^b^See supplementary material for a list of the data sources

### Data collection and analysis

In January 2014, we e-mailed birth defect program contacts from the 50 states, District of Columbia (DC), Puerto Rico (PR), Centers for Disease Control and Prevention (CDC), and U.S. Department of Defense Birth and Infant Health Registry (DOD) (*n* = 54) inviting them to complete the NBDPN Standards Data Quality Assessment Tool for their population-based birth defects programs. Follow-up e-mails and phone calls were conducted to remind programs to complete the tool. The completed Adobe Acrobat forms were electronically submitted to CDC for processing. Information submitted was exported to SAS 9.3 (Cary, NC) for analyses.

Eligible programs had to conduct population-based birth defect surveillance using active or passive case finding. A birth defects surveillance program was considered an active case-finding program if staff visit case identification sources (medical record abstraction) to collect original data on birth defects cases, and a passive case-finding program if the surveillance program relies on administrative databases or hospital reporting to identify birth defects cases with or without case verification.

To be included in the analyses a program must have met level 1 for data quality measure 1.1 [types of data sources used to identify potential cases] and have achieved an overall average score on all measures of at least 1. We calculated the average performance score for each measure and for the data quality attributes (completeness, timeliness, and accuracy) overall and by case-finding approach for each participating program.

## Results

Of the 50 states and four other programs in the United States that could potentially complete the Data Quality Assessment tool, 47 had eligible population-based birth defects surveillance programs. Of these, 45 programs submitted their completed assessment tool (96 % participation), but information from two programs were excluded since they did not meet minimum inclusion criteria. Therefore, the final analysis included information from 43 programs. The 17 programs categorized as using an active case-finding approach were: Arizona, Arkansas, California, Delaware, Georgia (Metropolitan Atlanta surveillance program operated by the CDC), Hawaii, Iowa, Louisiana, Massachusetts, Minnesota, New Hampshire, North Carolina, Oklahoma, Puerto Rico, South Carolina, Texas, and Utah. The 26 programs categorized as using passive case-finding were: Alaska, Colorado, Connecticut, DOD, Florida, Georgia (full state, operated by Georgia Department of Health), Illinois, Indiana, Kansas, Kentucky, Maine, Maryland, Michigan, Mississippi, Nebraska, New Jersey, New Mexico, New York, North Dakota, Ohio, Oregon, Rhode Island, Vermont, Virginia, West Virginia, and Wisconsin.

**Fig. 1:**
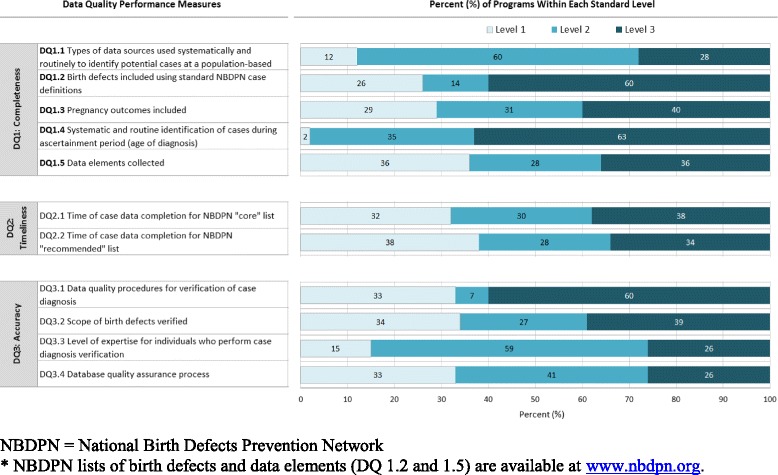
Distribution of score by data quality (DQ) standard level for each performance measure (*n* = 43)

The average scores for each NBDPN data quality measure for all 43 birth defects surveillance programs combined are presented in Table 2. The average score for each completeness performance measure was above level 2, except for data quality measure 1.5 (data elements collected). The average scores for both timeliness performance measures were below level 2, as were those for each accuracy measure, except for data quality measure 3.1 (data quality procedures for verification of case diagnosis). Three performance measures for which more than 75 % of the surveillance programs achieved at least a level 2 included: data quality measure 1.1 (types of data sources), 1.4 (systematic and routine identification of cases), and 3.3 (level of expertise for individuals who perform case diagnosis verification). The distribution of the scores for each data quality performance measure by self-reported standard level for all participating programs is displayed in Fig. 1.

**Table 2:** Average scores for completeness, timeliness, and accuracy overall and by program case-finding methodology

Data Quality (DQ) Performance Measures		Overall - All Programs, *n* = 43	Programs with Active Case-finding Methodology, *n* = 17	Programs with Passive Case-finding Methodology, *n* = 26
DQ1: Completeness	DQ1.1 Types of data sources used systematically and routinely to identify potential cases at a population-based level	2.2	2.5	1.9
	DQ1.2 Birth defects included using standard National Birth Defects Prevention Network case definitions ^a^	2.4	2.4	2.4
	DQ1.3 Pregnancy outcomes included	2.1	2.7	1.8
	DQ1.4 Systematic and routine identification of cases during ascertainment period (age of diagnosis)	2.6	2.5	2.7
	DQ1.5 Data elements collected ^a^	1.8	2.0	1.6
	Overall Completeness	2.2	2.4	2.1
DQ2: Timeliness	DQ2.1 Time of case data completion for NBDPN “core” list	1.8	1.7	1.9
	DQ2.2 Time of case data completion for NBDPN “recommended” list	1.5	1.4	1.5
	Overall Timeliness	1.6	1.5	1.7
DQ3: Accuracy	DQ3.1 Data quality procedures for verification of case diagnosis	2.3	2.9	1.9
	DQ3.2 Scope of birth defects verified	1.9	2.6	1.5
	DQ3.3 Level of expertise for individuals who perform case diagnosis verification	1.9	2.5	1.5
	DQ3.4 Database quality assurance process	1.7	2.1	1.5
	Overall Accuracy	2.0	2.5	1.6
	Overall (All Measures)	2.0	2.3	1.8

^a^NBDPN list of birth defects and data elements are available at www.nbdpn.org

The average scores by program case-finding approach for completeness, timeliness, and accuracy are also presented in Table 2. Surveillance programs using an active case-finding approach, on the average, scored higher than programs using a passive case-finding approach for the completeness (2.4 vs. 2.1) and accuracy (2.5 vs. 1.6) data quality performance measures, whereas their average score was lower for timeliness measures (1.5 vs. 1.7).

## Discussion

This is the first systematic attempt in the United States to comprehensively assess data quality (completeness, timeliness, and accuracy) across birth defects surveillance programs and provide information about areas for improvement. Our a priori expectation was that most programs would be able to achieve level 2 on all measures. In fact, the average score for each completeness performance measure was above level 2, except for data quality measure 1.5 (data elements collected). However, the average score for both timeliness performance measures were below level 2, as were those for all but one accuracy performance measure. Although all three data quality attributes are important, we recognize the tradeoffs that programs might need to make to focus on certain attributes instead of others given resource constraints. Further, because birth defects surveillance programs across the United States differ in methods, case ascertainment, purpose, and resources, our standards were designed to measure both the adequacy of methods in place, as well as data quality. Our findings demonstrated that all programs can enhance what they do and that challenges were different for programs that used active versus passive case-finding approaches; however, in general, data quality was highest for those programs with active case-finding approaches.

Other surveillance programs have established data quality standards and demonstrated the value of such improvements. Cancer registries in the United States developed a system for setting cancer surveillance standards and for assessing the quality, completeness, and timeliness of population-based cancer registries. The result is a system of certification for cancer registries that employs accepted measurable data assessments, routine methods for the auditing registry operations, and in the end, the ability to annually and efficiently assemble complete information on cancer incidence across the United States and its territories, as well as the provinces and territories in Canada.

Data collection for U.S. vital statistics has had a long history of national coordination. Standardized forms were first developed in 1900, and model vital records reporting statutes were first produced in 1907. Additionally, the National Association of Public Health Statistics and Information Systems (NAPHSIS), an organization of state health statistics offices, has worked to develop uniformity and comparability in health statistics information since 1933. These national efforts have translated into four decades of comparable vital statistics data within and across states that are routinely combined nationally to monitor population changes, assess population health, set and monitor progress in state and local public health initiatives, and facilitate research.

Similarly, the European Surveillance of Congenital Anomalies (EUROCAT) network of population-based birth defect registries developed a set of data quality indicators to assess completeness of case ascertainment, accuracy of diagnosis, completeness of information on EUROCAT variables, timeliness of data transmission, and availability of population denominator information [13]. EUROCAT and the NBDPN are assessing similar aspects of surveillance program data. However, our approach differs from EUROCAT in that they use more statistical metrics (i.e., percents, prevalences, ratios), while we are measuring approaches and methods that underlie these quantitative evaluations. As an example, EUROCAT uses neural tube defect prevalence and spina bifida-to-anencephaly ratio to measure under-ascertainment of birth defects cases among terminations of pregnancy. The NBDPN tool assesses which pregnancy outcomes the surveillance program includes. Thus, the NBPDN strategy is more qualitative and seeks to encourage the uniformity of methods across programs as a first step in moving towards consistent, high quality birth defects surveillance data in the United States.

The strengths of our effort include its high participation rate and the scope of input received in advance of our rollout of the data quality assessment tool. Almost one-half of the population-based birth defects surveillance programs in the United States participated in piloting the assessment tool and provided feedback to our workgroup. In addition, the standards were built on the foundation of existing NBDPN birth defects surveillance guidelines. Establishing guidelines was a necessary and effective first phase in developing standards for our programs; moving from guidelines to standards has been gradual. We now need to transition from guidelines to national standards to increase the quality, comparability, and utility of birth defects surveillance data. Finally, based on the high participation rate and feedback from programs, birth defects surveillance programs are interested and see the value of this initiative.

Our preliminary data are limited due to collection by self-assessment with no evaluation component. In addition, as this was our first U.S. assessment of birth defects surveillance programs, the tool and process continue to be improved; thus, these data serve as a preliminary baseline against which future data collections can be compared. Another limitation is that the data currently being collected to assess a surveillance program are primarily an accounting of registry practices and procedures and not an actual assessment of data quality and completeness. More formal surveillance system assessments typically examine these program attributes.

The development of national standards for birth defects in the United States remains a work in-progress. We considered 2014 a transition year, in which both state programs and the NBDPN could examine how programs fare in meeting the standards and could implement improvements that will enable programs to achieve more optimal standards. Programs can take steps to improve their consistency with the NBDPN standards, put processes in place to assist with achieving national standards, and serve as champions raising awareness about the value of national standards including articulating the need for resources to achieve national standards. Together, the birth defects surveillance community, working through the NBDPN, can develop a uniform set of standards that can serve to promote uniformity and enable surveillance programs to work towards improving these programs. The long term benefit will be improved and more comparable national data which will allow us to take advantage of current and future opportunities to better understand birth defects in the U.S.

## Conclusions

National standards for birth defects surveillance programs will allow for better pooling and translation of surveillance data at the state, multi-state, and national levels, thereby increasing the potential of these data to inform critical public health questions. The progression from recommended guidelines to established standards for birth defects surveillance is a logical step in the evolution of birth defects surveillance programs and offers the potential to generate data that will be more current, complete and accurate, as well as more uniform across states. Currently, about one-half of the birth defects surveillance programs in the United States meet the essential level of performance. Concerted efforts and resources will be needed to achieve uniform high quality national data.

## Additional file

Additional file 1:
**NBDPN Standards Assessment Tool on Data Quality.** (DOCX 215 kb)

## Abbreviations

CDC: Centers for Disease Control and Prevention
CRAA: Cancer Registries Amendment Act
DC: District of Columbia
DOD: Department of Defense Birth and Infant Health Registry
EUROCAT: European Surveillance of Congenital Anomalies
NAACCR: North American Association of Central Cancer Registries
NAPHSIS: National Association of Public Health Statistics and Information Systems
NBDPN: National Birth Defects Prevention Network
NCHS: National Center for Health Statistics
NCI: National Cancer Institute
NPCR: National Program of Cancer Registries
PR: Puerto Rico
US: United States
VSCP: Vital Statistics Cooperative Program
