# Supplementary material for: Development and implementation of the first national data quality standards for population-based birth defects surveillance programs in the United States
Source: BMC Public Health. 2015 Sep 19;15:925. doi: 10.1186/s12889-015-2223-2 (PMC4575466; doi:10.1186/s12889-015-2223-2)
Supplement: Additional file 1: — NBDPN Standards Assessment Tool on Data Quality. (DOCX 215 kb) [file 12889_2015_2223_MOESM1_ESM.docx]

Additional file 1: NBDPN Standards Assessment Tool on Data Quality

**National Birth Defects Prevention Network (NBDPN) Standards for Birth Defects Surveillance**

**Data Quality Assessment Tool**

2014 Version

Purpose: Performance standards for birth defects surveillance are intended to improve and standardize operations, outcomes, and surveillance functions across state programs, thereby making data more consistent and useful for a variety of purposes at local, state, multi-state, and national levels.

Format: This Assessment Tool lists performance indicators and associated measurements for data quality. Each line item measurement identifies the level of standards performance as (1) (2) or (3). Performance indicators are organized into completeness, timeliness and accuracy categories.

Definitions - explanations: Each performance indicator is followed by a definition that provides clarification of how to interpret the indicator and the reason/explanation for the specific performance indicator.

NBDPN Guidelines: These standards (i.e. performance indicator (and measurements)) will be linked directly back to the NBDPN Birth Defects Surveillance Guidelines and Standards Manual so that programs will understand how the guidelines and standards fit together (<http://www.nbdpn.org/birth_defects_surveillance_gui.php>). Chapters of the Manual that relate to each indicator are specified after the definition. Each performance measure references one or more chapters from the NBDPN Birth Defects Surveillance Guidelines and Standards. The applicable chapters include: Chapter 3 - Case Definition; Chapter 4 - Data Elements; Chapter 5 - Classification and Coding; Chapter 6 - Case Ascertainment Methods; Chapter 7 - Data Quality Management; Chapter 9 - Data Management and Security; and Chapter 12 - Inclusion of Prenatal Diagnoses in Birth Defects Surveillance.

Instructions: This is designed as a self-assessment tool for a birth defects surveillance system. Three performance levels are associated with each indicator:

Level 1: Rudimentary level of performance by a surveillance program

Level 2: Essential level of performance by a surveillance program

Level 3: Optimal level of performance by a surveillance program

Before completing each indicator, be sure to read all performance options.

**For each indicator, please check the highest performance level that applies for your birth defects surveillance system.**

**Before you start, please complete the identification box below.**

| State |  |
| --- | --- |
| Name of person completing tool |  |
| Title |  |
| Email |  |
| Date |  |

**Table of Contents**

[DQ1: Completeness 3](#_Toc355357983)

[DQ1.1 Types of data sources used systematically and routinely to identify potential cases at a population-based level 3](#_Toc355357984)

[DQ1.2 Birth defects included using standard NBDPN case definitions 4](#_Toc355357985)

[DQ1.3 Pregnancy outcomes included 4](#_Toc355357986)

[DQ1.4 Systematic and routine identification of cases during ascertainment period (age of diagnosis) 5](#_Toc355357987)

[DQ1.5 Data elements collected 5](#_Toc355357988)

[DQ2: Timeliness 6](#_Toc355357989)

[DQ2.1 Time of case data completion for NBDPN "core" list 6](#_Toc355357990)

[DQ2.2 Time of case data completion for NBDPN "recommended" list 7](#_Toc355357991)

[DQ3: Accuracy 8](#_Toc355357992)

[DQ3.1 Data quality procedures for verification of case diagnosis 9](#_Toc355357993)

[DQ3.2 Scope of birth defects verified 9](#_Toc355357994)

[DQ3.3 Level of expertise for individuals who perform case diagnosis verification 8](#_Toc355357994)

[DQ3.4 Database quality assurance process 9](#_Toc355357995)

**DQ1: Completeness**

Completeness is the extent to which data are all-inclusive and comprehensive. For example, are all of the cases of birth defects that occur within the target population, within a specified time period, identified by the surveillance system? (Reference: Chapter 7)

| Completeness **DQ1.1** | **DQ1.1 Types of data sources used systematically and routinely to identify potential cases at a population-based level**  This indicator is measuring the completeness of case ascertainment, and to some extent, accuracy of the program's surveillance data. The ultimate goal is to cast a wide net to ascertain as many cases as possible.  Reference: Chapters 6 and 12  **Please check the highest performance level that applies.** | | **Level 1** | **Level 2** | **Level 3** |
| --- | --- | --- | --- | --- | --- |
|  | **None or Unable to achieve level 1** (Please explain in comment box ) | **** |  |  |  |
|  | **Level 1**  Each of the following sources:   - Vital record data (e.g. birth and death certificates, fetal death certificate/report) - Additional source for case identification (e.g. hospital discharge, disease index) | | **** |  |  |
|  | **Level 2**  The data sources in level 1 and any additional sources of natal or postnatal data  Examples include:   - Insurance/Payer (Medicaid, All Payer Claims Database, Children’s Health Insurance Program (CHIP), Health Maintenance Organization (HMO), etc.) - Birth defects-specific reports submitted regularly by catchment area hospitals Public Health Program (Newborn Metabolic Screening; Newborn Hearing Screening; Maternal Child Health, such as Children with Special Health Care Needs Program (CSHCN), etc.) - Laboratories for natal or postnatal case identification and confirmation - Clinics (General Pediatrics, Cardiology, Central Nervous System (CNS)-Neurology, Developmental, Genetics, Musculoskeletal, Orofacial, Orthopedics, Urology, etc.) - Pathology (Anatomical Autopsy, Surgical, Tissue, etc.) - Providers (Pediatrician, Genetic Counselor, etc.) | | | **** |  |
|  | **Level 3**  The data sources in level 1, any of the additional data sources in level 2 and routine reporting from any of the following data sources for systematic specialized ascertainment of prenatally diagnosed defects (assessment of prenatal laboratory results is not sufficient for level 3)  Examples include:   - Maternal Fetal Medicine Clinics - Other prenatal diagnostic facilities (e.g. standalone radiology center) - Outpatient prenatal care clinics and offices | | | | **** |
|  | Comments: | | | | |

| Completeness **DQ1.2** | **DQ1.2 Birth defects included using standard NBDPN case definitions**^1^  This indicator reflects the scope of medical conditions in the surveillance database   Reference: Chapter 3  **Please check the highest performance level that applies.** | | **Level 1** | **Level 2** | **Level 3** |
| --- | --- | --- | --- | --- | --- |
|  | **None or Unable to achieve level 1** (Please explain in comment box ) | **** |  |  |  |
|  | **Level 1**  All of the NBDPN “core” birth defects | | **** |  |  |
|  | **Level 2**  All of the NBDPN "recommended" birth defects | | | **** |  |
|  | **Level 3**  Major structural malformations beyond those birth defects identified on the NBDPN list | | | | **** |
|  | Comments:  **^1^** NBDPN list of birth defects and data elements are available at [www.nbdpn.org](http://www.nbdpn.org). | | | | |

| Completeness **DQ1.3** | **DQ1.3 Pregnancy outcomes included**  This indicator identifies the types of pregnancy outcome categories in the surveillance data base, regardless of data sources.  Reference: Chapters 3 and 12  **Please check the highest performance level that applies.** | | **Level 1** | **Level 2** | **Level 3** |
| --- | --- | --- | --- | --- | --- |
|  | **None or Unable to achieve level 1** (Please explain in comment box ) | **** |  |  |  |
|  | **Level 1**  Live births | | **** |  |  |
|  | **Level 2**  Live births, stillbirths^1^ (fetal deaths at ≥20 weeks EGA^2^ OR >350 grams if EGA is not available) | | | **** |  |
|  | **Level 3**  Live births, stillbirths^1^ (fetal deaths at ≥20 weeks EGA^2^ OR >350 grams if EGA is not available), and other pregnancy loss, e.g. induced terminations | | | | **** |
|  | Comments:  ^1^ Programs may identify stillbirths from a variety of data sources, e.g. death certificates, hospital reports, pathology reports, etc.  ^2^EGA = estimated gestational age. Gestational age may be derived in various ways (including last menstrual period, physician prenatal estimate, postnatal exam, etc.). The NBDPN Birth Defects Surveillance Guidelines and Standards Chapter 3 gives a hierarchy of the accuracy of these methods. Programs should employ this hierarchy to use the most accurate method for including EGA in surveillance data. | | | | |

| Completeness **DQ1.4** | **DQ1.4 Systematic and routine identification of cases during ascertainment period (age of diagnosis)**  This is an indication of what is actually done (systematic and routine), not just the capacity/authority to do it.  Reference: Chapter 3  **Please check the highest performance level that applies.** | | **Level 1** | **Level 2** | **Level 3** |
| --- | --- | --- | --- | --- | --- |
|  | **None or Unable to achieve level 1** (Please explain in comment box ) | **** |  |  |  |
|  | **Level 1**  Identification of cases diagnosed through 1 month of age | | **** |  |  |
|  | **Level 2**  Identification of cases diagnosed through 1 year of age | | | **** |  |
|  | **Level 3**  Identification of cases diagnosed beyond 1 year of age | | | | **** |
|  | Comments: | | | | |

| Completeness **DQ1.5** | **DQ1.5 Data elements collected^1^**  A surveillance program should collect those items needed to fulfill its stated objectives. The value of the data is increased based on the extent to which data elements use clear definitions and are collected in a standardized way.  Reference: Chapter 4  **Please check the highest performance level that applies.** | | **Level 1** | **Level 2** | **Level 3** |
| --- | --- | --- | --- | --- | --- |
|  | **None or Unable to achieve level 1** (Please explain in comment box ) | **** |  |  |  |
|  | **Level 1**  All “core” data elements | | **** |  |  |
|  | **Level 2**  All “recommended” data elements | | | **** |  |
|  | **Level 3**  All “enhanced” data elements | | | | **** |
|  | Comments:  **^1^** NBDPN list of birth defects and data elements are available at [www.nbdpn.org](http://www.nbdpn.org). | | | | |

**DQ2: Timeliness**

Timeliness is the extent to which data are rapid, prompt, and responsive. For example, a birth defect case should be ascertained or reported to the program shortly after diagnosis. With rapid case identification the program is able to provide timely prevention and intervention services, respond quickly to investigations, and monitor trends. (Reference: Chapter 7)

| Timeliness **DQ2.1** | **DQ2.1 Time of case data completion for NBDPN "core" list**  Case identification to completion^1^, based on delivery year. Reflects when program regards data as “final”, and when the NBDPN could use these data in the annual report or a central data repository.  Reference: Chapters 3 and 7  **Please check the highest performance level that applies.** | | **Level 1** | **Level 2** | **Level 3** |
| --- | --- | --- | --- | --- | --- |
|  | **None or Unable to achieve level 1** (Please explain in comment box ) | **** |  |  |  |
|  | **Level 1**  ≥75% of all "core" NBDPN birth defects - reported cases complete within 2 years of delivery. | | **** |  |  |
|  | **Level 2**  ≥95% of all "core" NBDPN birth defects - reported cases complete within 2 years of delivery. | | | **** |  |
|  | **Level 3**  ≥99% of all "core" NBDPN birth defects - reported cases complete within 2 years of delivery. | | | | **** |
|  | Comments:  ^1^ “Complete” means the data are available for analysis/use and no further case investigation is required. | | | | |

| Timeliness **DQ2.2** | **DQ2.2 Time of case data completion for NBDPN "recommended" list**  Case identification to completion^1^, based on delivery year. Reflects when program regards data as “final”, and when the NBDPN could use these data in the annual report or a central data repository.  Reference: Chapters 3 and 7  **Please check the highest performance level that applies.** | | **Level 1** | **Level 2** | **Level 3** |
| --- | --- | --- | --- | --- | --- |
|  | **None or Unable to achieve level 1** (Please explain in comment box ) | **** |  |  |  |
|  | **Level 1**  ≥75% of all "recommended" NBDPN birth defects list-reported cases complete within 2 years of delivery. | | **** |  |  |
|  | **Level 2**  ≥95% of all "recommended" NBDPN birth defects list-reported cases complete within 2 years of delivery. | | | **** |  |
|  | **Level 3**  ≥99% of a "recommended" NBDPN birth defects list-reported cases complete within 2 years of delivery. | | | | **** |
|  | Comments:  ^1^ “Complete” means the data are available for analysis/use and no further case investigation is required. | | | | |

**DQ3: Accuracy**

Accuracy is the extent to which data are exact, correct and valid. For example, accurate diagnostic data affect a program's ability to provide reliable disease rates and to maintain data comparable to those from other programs. Diagnostic accuracy reflects the program's conformance with agreed-upon definitions and requirements. (Reference: Chapter 7)

| Accuracy **DQ3.1** | **DQ3.1 Data quality procedures for verification of case diagnosis**  This indicator includes ongoing data quality procedures for accuracy and completeness of the case diagnosis.  Verification of case diagnosis is an important quality assurance procedure. Quality assurance procedures should be conducted as specified in the guidelines manual.  Reference: Chapter 7  **Please check the highest performance level that applies.** | | **Level 1** | **Level 2** | **Level 3** |
| --- | --- | --- | --- | --- | --- |
|  | **None or Unable to achieve level 1** (Please explain in comment box ) | **** |  |  |  |
|  | **Level 1**  Minimal data quality procedure for case verification, majority of cases accepted as reported | | **** |  |  |
|  | **Level 2**  Verification using “some” method, e.g. clinical case report from a specialty clinic, agreement across multiple data sources, agreement between procedure and diagnostic codes, laboratory reports | | | **** |  |
|  | **Level 3**  Verification using method beyond level 2, e.g. medical records | | | | **** |
|  | Comments: | | | | |
|  |  |  |  |  |  |

| Accuracy **DQ3.2** | **DQ3.2 Scope of birth defects verified**  This indicator includes ongoing data quality procedures for accuracy and completeness of the case diagnosis.  Reference: Chapter 7  **Please check the highest performance level that applies.** | | **Level 1** | **Level 2** | **Level 3** |
| --- | --- | --- | --- | --- | --- |
|  | **None or Unable to achieve level 1** (Please explain in comment box ) | **** |  |  |  |
|  | **Level 1**  Special projects, selected diagnoses, or samples only | | **** |  |  |
|  | **Level 2**  Verification for all “core” birth defects | | | **** |  |
|  | **Level 3**  Verification for all “recommended” birth defects | | | | **** |
|  | Comments: | | | | |
|  |  |  |  |  |  |

| Accuracy **DQ3.3** | **DQ3.3 Level of expertise for individuals who perform case diagnosis verification**  This will determine the extent to which a program has the capacity to determine diagnosis. Program staff should maintain a level of expertise, through on-going training.  Reference: Chapters 5, 6 and 7  **Please check the highest performance level that applies.** | | **Level 1** | **Level 2** | **Level 3** |
| --- | --- | --- | --- | --- | --- |
|  | **None or Unable to achieve level 1** (Please explain in comment box ) | **** |  |  |  |
|  | **Level 1**  Staff with no or minimal disease coding or clinical expertise perform routine case reviews | | **** |  |  |
|  | **Level 2**  Staff with expertise in disease coding or clinical training (e.g. RN or genetic counselor) perform routine case reviews | | | **** |  |
|  | **Level 3**  Clinical geneticist, dysmorphologist or other high level expert depending on defect (i.e. pediatric cardiologist for heart defects) routinely performs case reviews | | | | **** |
|  | Comments: | | | | |

| Accuracy **DQ3.4** | **DQ3.4 Database quality assurance process**  The quality of the registry database can be enhanced significantly through use of quality assurance steps during data collection and processing.  Procedures to screen the data for potential error at the field level, the record level and across records for an individual can enable isolating and resolving problems with the data.  Standardization of data elements is an important quality assurance procedure. Quality assurance procedures for each data element should be conducted as specified in the guidelines manual.  Reference: Chapters 4, 7 and 9  **Please check the highest performance level that applies.** | | **Level 1** | **Level 2** | **Level 3** |
| --- | --- | --- | --- | --- | --- |
|  | **None or Unable to achieve level 1** (Please explain in comment box ) | **** |  |  |  |
|  | **Level 1**  Quality checks are performed for “core” NBDPN data elements. | | **** |  |  |
|  | **Level 2**  Quality checks are performed for “recommended” NBDPN data elements. | | | **** |  |
|  | **Level 3**  Quality checks are performed for “enhanced” NBDPN data elements. | | | | **** |
|  | Comments: | | | | |

**Summary**

**[Note that you do not need to complete the following table. These scores will be generated automatically.]**

| Performance Measure | None | Level 1 | Level 2 | Level 3 |
| --- | --- | --- | --- | --- |
| DQ1: Completeness |  |  |  |  |
| DQ1.1 Types of data sources used systematically and routinely to identify potential cases at a population-based level |  |  |  |  |
| DQ1.2 Birth defects included using standard NBDPN case definitions |  |  |  |  |
| DQ1.3 Pregnancy outcomes included |  |  |  |  |
| DQ1.4 Systematic and routine identification of cases during ascertainment period (age of diagnosis) |  |  |  |  |
| DQ1.5 Data elements collected |  |  |  |  |
| DQ2: Timeliness |  |  |  |  |
| DQ2.1 Time of case data completion for NBDPN "core" list |  |  |  |  |
| DQ2.2 Time of case data completion for NBDPN "recommended" list |  |  |  |  |
| DQ3: Accuracy |  |  |  |  |
| DQ3.1 Data quality procedures for verification of case diagnosis |  |  |  |  |
| DQ3.2 Scope of birth defects verified |  |  |  |  |
| DQ3.3 Level of expertise for individuals who perform case diagnosis verification |  |  |  |  |
| DQ3.4 Database quality assurance process |  |  |  |  |
